# Yeast two-hybrid screening for proteins that interact with PFT in wheat

**DOI:** 10.1038/s41598-019-52030-x

**Published:** 2019-10-29

**Authors:** Yi He, Lei Wu, Xiang Liu, Xu Zhang, Peng Jiang, Hongxiang Ma

**Affiliations:** 1 Institute of Food Crops, Jiangsu Academy of Agricultural Sciences/Jiangsu Collaborative Innovation Center for Modern Crop Production, Nanjing, China; 2 Tibet Agriculture and Animal Husbandry University, Linzhi, China

**Keywords:** Biotechnology, Plant sciences

## Abstract

*Fusarium* head blight (FHB) is a devastating disease of wheat worldwide. *Fhb1* is the most consistently reported quantitative trait locus (QTL) for FHB resistance breeding. A pore-forming toxin-like (*PFT*) gene at *Fhb1* was first cloned by map-based cloning and found to confer FHB resistance in wheat. Proteins often interact with each other to execute their functions. Characterization of the proteins interacting with PFT might therefore provide information on the molecular mechanisms of PFT functions. In this study, a high-quality yeast two-hybrid (Y2H) library using RNA extracted from *Fusarium graminearum* (*Fg*)-infected wheat spikes of Sumai 3 was constructed. The agglutinin domains of PFT exhibited no self-activation and toxicity to yeast cells and were used as bait to screen the Y2H library. Twenty-three proteins that interact with PFT were obtained, which were mainly involved in the ubiquitination process, clathrin coat assembly, the oxidation-reduction process, and protein phosphorylation. The expression pattern of these interacting genes was analyzed by quantitative real-time PCR. This study clarifies the protein interactions of PFT and raises a regulatory network for PFT regarding FHB resistance in wheat.

## Introduction

Wheat is one of the world’s most important food crops, and demand for wheat is increasing due to the growth of the human population. However, wheat production is facing continuous threat from different biotic and abiotic stress factors. Globally, *Fusarium* head blight (FHB) is one of the most destructive fungal diseases of wheat. The disease not only results in heavy yield losses but also reduces grain quality by producing harmful deoxynivalenol and other trichothecene toxins posing a risk to global food security^1,2^.

Development of FHB-resistant cultivars is an effective approach to reduce damage from FHB. However, FHB resistance in wheat is an overly complex quantitative trait controlled by multiple quantitative trait loci (QTLs) with significant genotype–environment interactions^3–5^. Over 100 QTLs associated with FHB resistance have been reported on all 21 chromosomes of wheat^5^. Among these, *Fhb1*, identified in Sumai 3 and other Chinese cultivars, is the strongest and best validated resistance QTL^6,7^. Laborious and continuous studies have been carried out to identify candidate genes in *Fhb1* by various approaches, including transcriptome-based analysis and genomic contig sequencing^8,9^. Functional validation of candidate genes in wheat remained elusive until pore-forming toxin-like (PFT) in the *Fhb1* region was reported to confer FHB resistance by mutation analysis, gene silencing, and transgenic overexpression in wheat^10^. *PFT* is predicted to encode a chimeric lectin with two agglutinin domains and an ETX/MTX2 toxin domain; further studies are needed to understand the mechanism underlying PFT action.

Protein–protein interaction networks are important sources of information related to biological processes and complex metabolic functions in living cells. The yeast two-hybrid (Y2H) system, originally developed by Field and Song, is one of the most practical tools to identify the interacting partners of proteins in regulatory complexes^11^. The Y2H system exploits eukaryotic transcriptional activators containing separable functional domains for DNA-binding and transactivation, and it represents a powerful and fast approach to identify new protein interacting partners for a protein of interest that has been widely used in various organisms, including plants, animals, and fungi. Several wheat proteins in the regulatory network have been revealed using the Y2H screen system. The key vernalization gene *TaVRN-A1* was used as bait to elucidate interacting proteins in wheat^12^. A cytosolic glutamine synthetase GS1b has been found to interact with the mitogen-activated protein kinase tMEK2 in plant defence against pathogen attacks, by Y2H screening^13^. The proteins encoded by the photosynthesis-related genes *PSK-I* and *PsbS1* have been confirmed to interact with the truncated haemoglobin protein trHb in photosynthesis^14^. These proteins are good entry points to further elucidate their regulatory network in the development process.

In this study, to screen proteins that interact with PFT, a high-quality Y2H library using *Fusarium graminearum* (*Fg*)-infected wheat spikes of the FHB-resistant cultivar Sumai 3 was constructed. Using the Y2H screening system, we screened some proteins that interact with PFT and provide information on the proteins interacting with PFT that are putatively involved in FHB resistance in wheat.

## Results

### Evaluation of the effect of yeast growth

**Figure 1:**
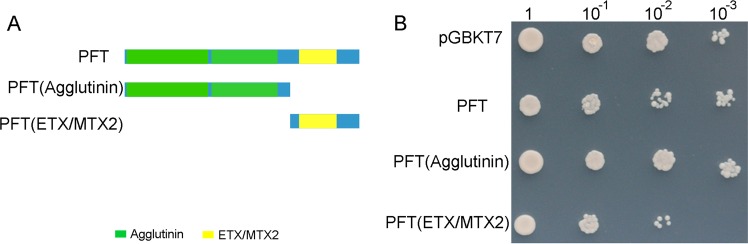
Effect of PFT and its two domains on the growth of *S*. *cerevisiae*. (**A**) Schematic presentation of the PFT domains and deletion constructs used to transform the yeast cells. (**B**) The full-length PFT, its two domains, and pGBKT7 used as the control, were separately transformed into the *S*. *cerevisiae* strain Y2HGold. The transformants were plated on synthetically defined (SD) medium lacking tryptophan (SD/-Trp), and the ETX/MTX2 domain had a toxic effect on yeast growth.

PFT contains a chimeric lectin with two agglutinin domains and an ETX/MTX2 toxin domain. The ETX/MTX2 proteins are potent toxins posing potential threats to living cells^10^. To test whether PFT is toxic to *Saccharomyces cerevisiae* (yeast), the full-length PFT and its two domains were separately transformed into the Y2HGold yeast strain, and then plated on synthetically defined (SD) medium lacking leucine (SD/-Leu) medium (Fig. 1A). The growth of yeast cells containing only the agglutinin domains was not affected, but the expression of the ETX/MTX2 toxin domain had a toxic effect on yeast growth compared with that of the empty vector control (Fig. 1B).

### Confirmation of bait autoactivation

**Figure 2:**
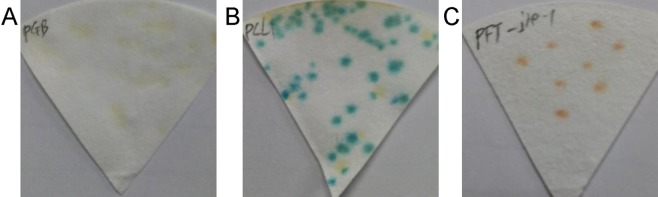
β-galactosidase assay indicating no self-activation of PFTa. The Y190 yeast cells that were transformed with negative control pGBKT7 vectors (**A**), positive control PCL1 (which encodes full-length wild-type GAL4) (**B**), and pGBKT7-PFTa (which contains only the agglutinin domains) (**C**), were plated on synthetically defined (SD) medium lacking tryptophan but containing X-Gal (SD/-Trp/X-Gal) for the autoactivation test. The yeast transfected with plasmids pGBKT7 (**A**) and pGBKT7-PFTa (**C**) did not turn blue in the β-galactosidase assay, indicating that pGBKT7-PFTa does not autonomously activate the reporter genes in yeast cells without a prey protein.

The expression of the full-length PFT had a negligible toxic effect on yeast growth; therefore, we chose the partial PFT containing only the agglutinin domains (PFTa) to further analyze protein–protein interactions. The vectors pGBKT7, pCL1, and pGBKT7-PFTa were transformed into the Y190 yeast strain, and then the resulting transformants were plated on SD medium lacking tryptophan but containing a chromogenic substrate for yeast galactosidase (SD/-Trp/X-α-Gal). The positive control pCL1 colonies turned blue on SD/-Trp/X-α-Gal plate (Fig. 2A), but like the negative control, pGBKT7-PFTa colonies did not turn blue (Fig. 2B,C), indicating that the bait pGBKT7-PFTa could not autonomously activate the reporter genes in the absence of prey protein and was, therefore, suitable for screening the Y2H library.

### Construction of the Y2H library

**Figure 3:**
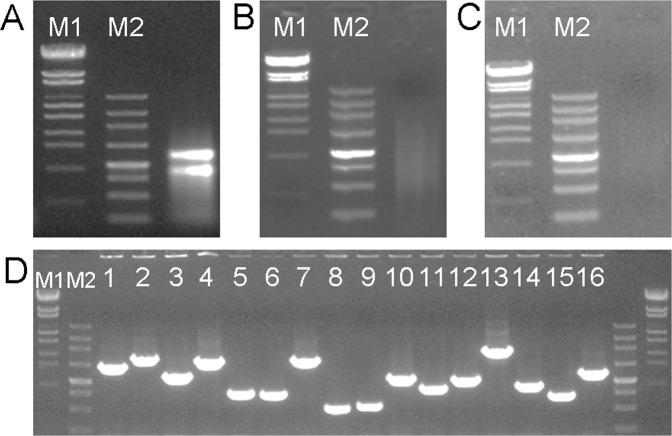
The Y2H library construction and quality test. The integrity of total RNA from the *Fg*-treated spikes of Sumai 3 after DNase I digestion (**A**) and the ds cDNA after normalization were evaluated by 1% agarose gel electrophoresis (**B**). (**C**) The fragments smaller than 200 bp were eliminated after purification using a Chroma spin-1000 column. (**D**) The PCR products of 16 clones showed that the bands of cDNA fragments ranged from 0.5 to 2.25 kb in size. M1, Lambda EcoT14I digest marker (Takara, Cat. No. 3401); M2, 250 bp DNA ladder marker (Takara, Cat. No. 3424).

Total RNA from the spike of Sumai 3 after *Fg* inoculation was used for the construction of the Y2H library. The integrity of total RNA after DNaseI digestion was identified by electrophoresis on a 1% agarose gel. Several bands corresponding to ribosomal 28 S and 18 S were observed (Fig. 3A), indicating that the total RNA was complete and did not degrade. To obtain a high-quality cDNA for the subsequent library construction, the cDNA was synthesized using the SMART technology (Clontech). The synthesized cDNA was then normalized and analyzed by electrophoresis on a 1% agarose gel. The bands of cDNA fragments ranged from 0.5 to 2.25 kb in size (Fig. 3B). Small cDNA fragments were eliminated after purification using a Chroma Spin-1000 column (Fig. 3C), and then transferred to the pGADT7 vector to construct a Y2H library. The transformation reaction mixture was diluted from 1:10 to 1:1000, and then spread on LB plates to calculate transformation efficiency. The Y2H library had ~2 × 10^6^ total primary clones and the final library titre was 1.0 × 10^9^ cfu/mL, which far exceeded the minimum cell density, 2 × 10^7^ cells/mL, required for Y2H screening. To determine the length of the inserts of the cDNA library, 16 positive clones were randomly selected and identified by polymerase chain reaction (PCR). The results showed that the inserted fragments ranged from 0.5 to 2.25 kb in size (Fig. 3D). These findings indicated that the Y2H library could be used for further research.

### Screening of PFT-interacting proteins

**Figure 4:**
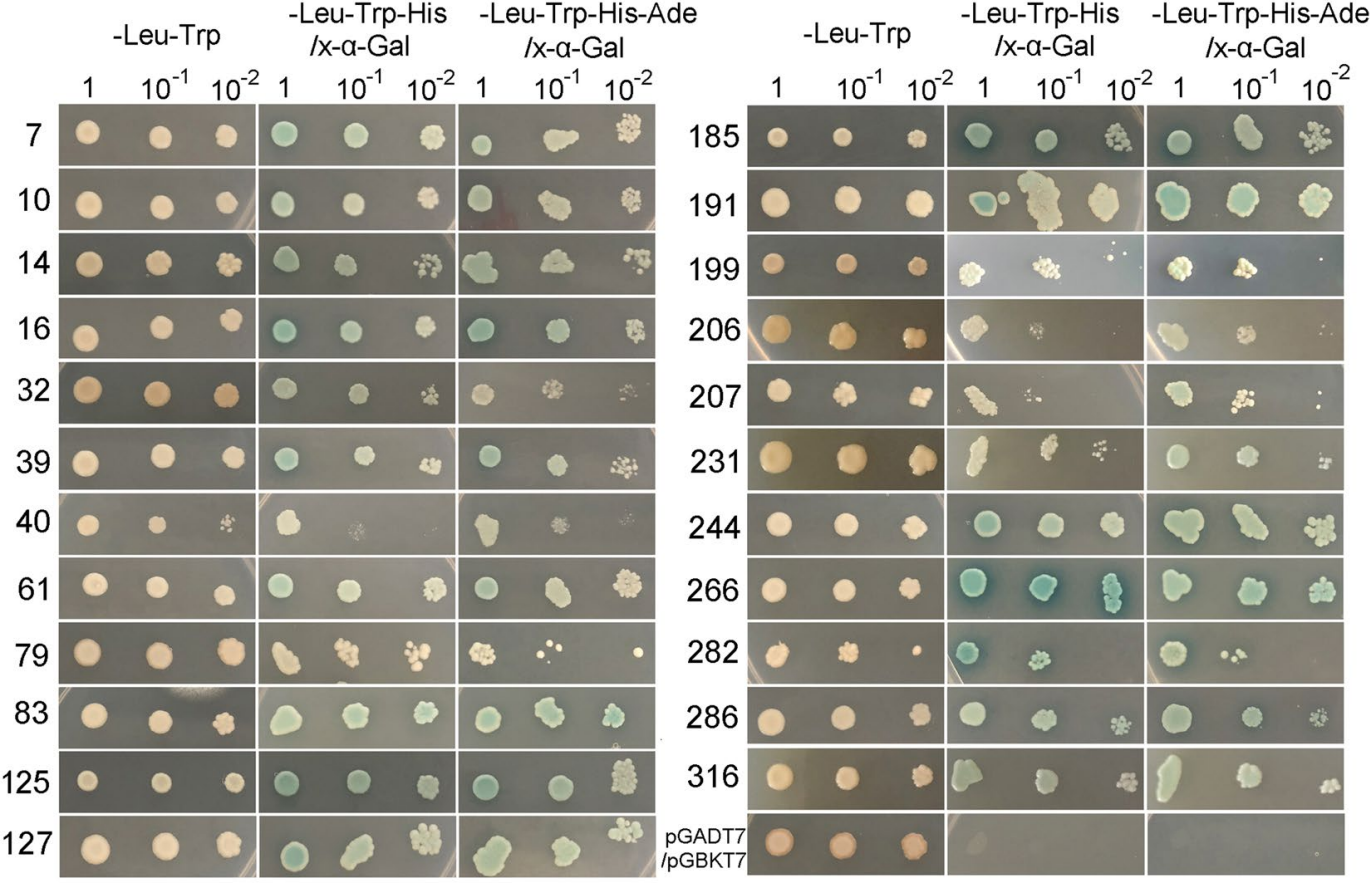
Confirmation of the interaction between PFTa and the 23 proteins by Y2H assays. The pGBKT7-PFTa together with each of the 23 prey plasmids were transformed into Y2HGold yeast cells individually. The transformants were then plated on synthetically defined (SD) medium lacking leucin (Leu) and tryptophan (Trp) (SD/-Leu/-Trp), on SD medium lacking Leu, Trp, and histidine (His) but containing X-α-Gal (SD/-Leu/-Trp/-His/X-α-Gal), and on SD/-Leu/-Trp/-His/X-α-Gal medium lacking adenine (SD/-Leu/-Trp/-His/-Ade/X-α-Gal), and cultivated for 3–5 days at 30 °C. Positive interaction was indicated by the presence of blue colonies. Co-transformation with pGADT7 and pGBKT7 was used as a negative control.

The transformants expressing interacting pairs of pGBKT7-PFTa and AD library proteins in Y190 yeast cells were incubated on SD medium lacking Trp, Leu, and histidine but containing 30 mM 3-amino-1,2,4-triazole (SD/-Trp/-Leu/-His + 30 mM 3-AT) for the first screening. A total of 212 clones grew out. Plasmids from all of them were isolated and sequenced and 57 different genes were identified. As there were false positive clones, we transformed the plasmid pGBKT7-PFTa and the 57 plasmids representing different genes into Y2HGold yeast cells individually. The Y2HGold strain is unable to synthesize His and adenine (Ade) and it is therefore unable to grow on media that lack either of the two essential amino acids. When bait and prey proteins interact, Gal4-responsive His3 and Ade2 expression allow the cell to biosynthesize both of these amino acids, and grow on –His–Ade minimal medium. The cotransformants were then grown on SD/-Trp/-Leu/-His/X-α-Gal and SD/-Trp/-Leu/-His/-Ade/X-α-Gal media. Finally, 23 positive clones representing different genes were identified through screening (Fig. 4).

### Identification of proteins interacting with PFT

The 23 positive clones were further analyzed using the basic local alignment search tool (BLAST) of the National Center for Biotechnology Information (NCBI) and UniProt databases (Table 1). Among them, only five proteins were characterized, namely, an acetolactate synthase (A0A3B6QKH3), a proteasome subunit beta (W5GEL1), a superoxide dismutase (Q96185), a mitogen-activated protein kinase (A0A3B5ZVC6), and a serine/threonine-protein phosphatase (A0A3B6MZU1). Five other proteins A0A3B6EIL3, A0A3B6NP03, A0A3B6AYL3, A0A3B6GUJ7, and A0A3B5YVG0, presented the highest screening frequency with 118 appearances, covering 55.6% of the 212 clones. To obtain more information on these proteins, we analyzed their homology function in the wheat species *Aegilops tauschii* subsp. *tauschii* (Table 1). Proteins related to DNA/RNA binding were the most frequent, including A0A3B6GUJ7, A0A3B6TJ86, A0A3B5XY63, A0A3B6DMV5, and A0A3B6INR9. Proteins related to the ubiquitination pathway were also frequent, including A0A3B6EIL3, A0A3B5YVG0, and W5GEL1. A protein with superoxide dismutase activity named Q96185 was also found. The function of these interacting proteins with PFT was further subject to gene ontology (GO) analysis (Table 2). These proteins participated in the regulation of transcription, proteolysis, the oxidation-reduction process, protein phosphorylation, and clathrin coat assembly. Protein domain architectures provide useful information to determine protein functions. We further analyzed domains of the 23 interacting proteins using the SMART and Pfam databases. Consistent with the above homology function and GO analysis, diverse domains were found in these proteins including zinc finger (Znf), RING domain, ENTH domain, DDT domain, PP2A, serine/threonine protein kinase (S_TKc) domain, and RRM domain (Supplementary Fig. S1).

**Table 1:** The information of the genes interacting with PFT.

No.	Gene Name	UniProtKB Name	Protein Name	Appearance	Homology Function in *Aegilops tauschii* subsp. *tauschii*
7	TraesCS3A02G288900	A0A3B6EIL3	Uncharacterized protein	46	E3 ubiquitin-protein ligase MIEL1-like
10	TraesCS1B02G159900	A0A3B5YVG0	Uncharacterized protein	10	E3 ubiquitin-protein ligase MIEL1-like
14	TraesCS6D02G268700	A0A3B6QKH3	Acetolactate synthase	1	Acetolactate synthase 1
16	TraesCS2A02G295900	A0A3B6AYL3	Uncharacterized protein	16	OTU domain-containing protein 5-B
32	TraesCS6B02G174200	W5GEL1	Proteasome subunit beta	1	Proteasome subunit beta type-3-like
39	TraesCS4D02G021400	A0A3B6JDL9	Uncharacterized protein	6	Phosphomethylpyrimidine synthase
40	TraesCS6A02G154100	A0A3B6NP03	Uncharacterized protein	31	Clathrin assembly protein
61	TraesCS3D02G245800	A0A3B6GUJ7	Uncharacterized protein	15	Homeobox-DDT domain protein RLT2-like
79	TraesCS2D02G538300	Q96185	Superoxide dismutase	1	Superoxide dismutase
83	TraesCS2D02G267600	A0A2X0S5V9	Uncharacterized protein	1	Uncharacterized protein
125	TraesCS2A02G191000	A0A3B6AV80	Uncharacterized protein	1	MO25-like protein
127	TraesCS1D02G192200	A0A3B5ZVC6	Mitogen-activated protein kinase	1	Mitogen-activated protein kinase 6
185	TraesCS7D02G270800	A0A3B6TJ86	Uncharacterized protein	1	Nuclear speckle RNA-binding protein A-like
191	TraesCS1B02G244100	A0A3B5YY23	Uncharacterized protein	1	Chaperone protein dnaJ 49
199	TraesCS1A02G150600	A0A3B5XY63	Uncharacterized protein	1	Heterogeneous nuclear ribonucleoprotein 1-like
206	TraesCS6B02G181800	A0A3B6PLA2	Uncharacterized protein	1	Clathrin assembly protein
207	TraesCS2D02G529100	A0A3B6DMV5	Uncharacterized protein	1	UBP1-associated protein 2B-like
231	TraesCS5A02G250100	A0A3B6KI95	Uncharacterized protein	1	Uncharacterized protein
244	TraesCS3D02G460200	A0A3B6H597	Uncharacterized protein	2	Tankyrase-1-like
266	TraesCS5D02G465400	A0A3B6MZU1	Serine/threonine-protein phosphatase	1	Serine/threonine-protein phosphatase PP2A-2 catalytic subunit
282	TraesCS4B02G153900	A0A3B6INR9	Uncharacterized protein	2	Polypyrimidine tract-binding protein homolog 1-like
286	TraesCS2A02G306800	A0A3B6AZH5	Uncharacterized protein	1	NAC domain-containing protein 104
316	TraesCS5B02G455800	A0A3B6LV94	Uncharacterized protein	1	Uncharacterized protein

**Table 2:** Gene ontology analysis on proteins interacting with PFT.

Gene Name	Molecular function	Biological process	Cellular component
TraesCS3A02G288900	GO:0008270 zinc ion binding		
TraesCS1B02G159900	GO:0008270 zinc ion binding	GO:1902456 regulation of stomatal opening	
TraesCS6D02G268700	GO:0003824 catalytic activity	GO:0008652 cellular amino acid biosynthetic process	GO:0009507 chloroplast
	GO:0003984 acetolactate synthase activity	GO:0009082 branched-chain amino acid biosynthetic process	GO:0009570 chloroplast stroma
	GO:0016740 transferase activity	GO:0009097 isoleucine biosynthetic process	
	GO:0016829 lyase activity	GO:0009099 valine biosynthetic process	
TraesCS2A02G295900	GO:0004843 thiol-dependent ubiquitin-specific protease activity	GO:0016579 protein deubiquitination	
	GO:0003682 chromatin binding	GO:0016578 histone deubiquitination	
	GO:0031491 nucleosome binding	GO:0045892 negative regulation of transcription, DNA-templated	
	GO:0042393 histone binding		
TraesCS6B02G174200	GO:0004175 endopeptidase activity	GO:0006508 proteolysis	GO:0000502 proteasome complex
	GO:0004298 threonine-type endopeptidase activity	GO:0043161 proteasome-mediated ubiquitin-dependent protein catabolic process	GO:0005634 nucleus
	GO:0008233 peptidase activity	GO:0051603 proteolysis involved in cellular protein catabolic process	GO:0005737 cytoplasm
	GO:0016787 hydrolase activity		GO:0005839 proteasome core complex
TraesCS4D02G021400	GO:0016830 carbon-carbon lyase activity	GO:0009228 thiamine biosynthetic process	GO:0009536 plastid
	GO:0019904 protein domain specific binding		GO:0009507 chloroplast
TraesCS6A02G154100	GO:0005543 phospholipid binding	GO:0048268 clathrin coat assembly	GO:0030136 clathrin-coated vesicle
	GO:0030276 clathrin binding		
TraesCS3D02G245800	GO:0003677 DNA binding	GO:0006355 regulation of transcription, DNA-templated	
TraesCS2D02G538300	GO:0004784 superoxide dismutase activity	GO:0019430 removal of superoxide radicals	
	GO:0016491 oxidoreductase activity	GO:0055114 oxidation-reduction process	
TraesCS1D02G192200	GO:0004672 protein kinase activity	GO:0000165 MAPK cascade	GO:0005622 intracellular
	GO:0000166 nucleotide binding	GO:0006468 protein phosphorylation	
	GO:0004707 MAP kinase activity	GO:0016310 phosphorylation	
	GO:0016301 kinase activity		
TraesCS7D02G270800	GO:0003723 RNA binding		
TraesCS1A02G150600	GO:0003723 RNA binding		
	GO:0003676 nucleic acid binding		
TraesCS6B02G181800	GO:0005543 phospholipid binding	GO:0048268 clathrin coat assembly	GO:0030136 clathrin-coated vesicle
	GO:0030276 clathrin binding		
TraesCS2D02G529100	GO:0003723 RNA binding		
	GO:0003676 nucleic acid binding		
TraesCS5A02G250100	GO:0003677 DNA binding		
	GO:0003676 nucleic acid binding		
TraesCS5D02G465400	GO:0016787 hydrolase activity	GO:0006470 protein dephosphorylation	
	GO:0004721 phosphoprotein phosphatase activity		
TraesCS4B02G153900	GO:0003723 RNA binding		
	GO:0003676 nucleic acid binding		
TraesCS2A02G306800	GO:0003677 DNA binding	GO:0006355 regulation of transcription, DNA-templated	
TraesCS5B02G455800	GO:0002151 G-quadruplex RNA binding		GO:0031011 Ino80 complex
	GO:0005515 protein binding		GO:0071339 MLL1 complex

### Expression profiles of the interacting genes

**Figure 5:**
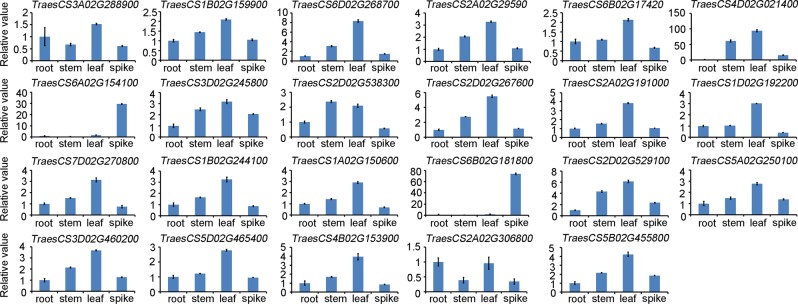
Different expression levels of the interacting genes in the root, stem, flag leaf, and spike of Sumai 3. The transcript levels of the 23 genes were analyzed by quantitative real-time PCR. The wheat *tubulin* gene was used as the endogenous control gene. The gene expression levels were expressed as relative values compared to the value in root, and error bars indicate mean ± standard error (SE) from the results of three replicates.

**Figure 6:**
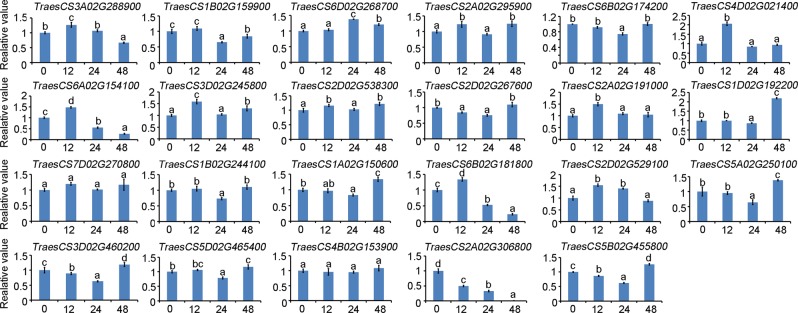
Different expression levels of the interacting genes in wheat spikes after *Fg* inoculation. Spikes of Sumai 3 at early anthesis were injected with 10 μl *Fg* suspension (1 × 10^6^ conidia/mL). The inoculated spikes were covered with plastic bags to maintain the moisture for fungal infection. Samples were collected at 0, 12 24 and 48 h after *Fg* inoculation. The wheat *tubulin* gene was used as the endogenous control gene. The gene expression levels were expressed as relative values compared to the value in non-*Fg* inoculated spikes, and were analyzed by two-way ANOVA with Tukey’s significant difference test. The values are means ± standard error (SE) from the results of three replicates, and significant differences are indicated by different lowercase letters (*p* < 0.05).

The transcript levels of *PFT* were low in the root, stem, and flag leaf, and high in the early developing spikes. *PFT* was also induced after *Fg* inoculation in Sumai 3^15^. When two proteins interact, they are more likely to be co-expressed^16^. To observe the expression profiles of the interacting genes in Sumai 3, we analyzed the relative expression of these genes in the root, stem, leaf, and spike. The graphical representation of the expression profiles of 23 interacting genes in the four tissues is shown in Fig. 5. Most of the interacting genes were highly expressed in the leaf. TraesCS6A02G154100 and TraesCS6B02G181800 were specially expressed in the spike. To identify whether the interacting genes were induced after *Fg* treatment, the expression levels of these genes after 12, 24, and 48 h of *Fg* inoculation were investigated. Eight genes, including TraesCS3A02G288900, TraesCS2A02G295900, TraesCS4D02G021400, TraesCS6A02G154100, TraesCS3D02G245800, TraesCS2A02G191000, TraesCS6B02G181800, and TraesCS2D02G529100 were up-regulated by at least 1.2-fold 12 h after *Fg* inoculation, while eight genes, including TraesCS6D02G268700, TraesCS2A02G295900, TraesCS3D02G245800, TraesCS2D02G538300, TraesCS1D02G192200, TraesCS1A02G150600, TraesCS5A02G250100 and TraesCS5B02G455800 were up-regulated by at least 1.2-fold 48 h after *Fg* inoculation (Fig. 6).

## Discussion

Proteins often work together with other partner proteins to accomplish their essential functions in living organisms. Thus, the determination of protein–protein interactions is helpful for elucidating the molecular functions of relevant protein molecules involved in plant development and responses to stresses. The *PFT* gene at *Fhb1* has been reported to confer FHB resistance in wheat, but the role of PFT in FHB resistance is controversial^10,17,18^. Although *PFT* was up-regulated after *Fg* inoculation, its expression pattern has been reported to differ among some resistant and susceptible varieties^15^. It is possible that the expression and function of *PFT* is influenced by other genes. Therefore, it is necessary to identify proteins that interact with PFT to understand its resistance mechanism. In this study, we constructed a high-quality Y2H library using RNA extracted from *Fg*-infected wheat spikes of the FHB-resistant cultivar Sumai 3, and performed Y2H screening to identify putative proteins that interact with PFT.

A high-quality Y2H library can provide molecular resources to analyze protein functions and interactions based on a known protein to facilitate the construction of protein networks^19^. These proteins provide baseline information for further elucidating their regulatory network in the development process. Library titre, recombination rate, and the size of inserted fragments indicated the quality of the cDNA library^20^. The library titre should be not less than 1.7 × 10^5^ cfu. Our primary library storage capacity was over 2.0 × 10^6^ cfu, the recombination rate was approximately 100%, and the final library titre was over 1.0 × 10^9^ cfu/mL. Clones were randomly selected and showed that the inserted fragments in our library were ranged from 0.5 to 2.25 kb, indicating that the Y2H library was of high quality and could therefore be used for further research.

PFT contains one ETX/MTX2 domain, which are potent bacterial toxins that form channels in the cell membrane of the host leading to cell death^10^. We then checked the effects of PFT on yeast growth using the full-length protein and its two domains separately. Although the effect was negligible at a high concentration (OD600 = 1) in all the transformed yeast strains, growth inhibition of the full-length PFT and its ETX/MTX2 domain-transformed yeast strains was observed at low concentrations (OD600 = 0.01 to 0.001), but not in agglutinin domain-transformed yeast strains. Agglutinin interacts with other proteins in various biological processes. The interactions of garlic (*Allium sativum*) leaf agglutinin with a chaperonin group of unique receptor protein isolated from a bacterial endosymbiont of mustard aphid have been identified in a symbionin-mediated virus transmission study^21^. Therefore, we chose the integrant PFT sequences containing only agglutinin domains for further analysis of protein–protein interaction, and this partial PFT was used as bait to screen the Y2H library in this study as it could not autonomously activate the reporter genes without prey protein.

In the present study, we identified 23 proteins that putatively interacted with PFT. Among them, five proteins presented the highest screening frequency with 118 appearances, covering 55.6% of the 212 clones. These five proteins were A0A3B6EIL3 (46), A0A3B6NP03 (31), A0A3B6AYL3 (16), A0A3B6GUJ7 (15), and A0A3B5YVG0 (10). Both A0A3B6EIL3 and A0A3B5YVG0 carry the Znf and RING domains. The Znf-containing proteins have pivotal functions in various processes of plant growth and development, as well as in resistance to biotic and abiotic stresses. Several disease-resistant genes such as *Pib* (rice), *Lr10* (wheat), *RCY1* (Arabidopsis), and *Rpg1* (barley) contain multiple numbers of Znf domains^22^. Numerous RING-containing proteins have ubiquitin ligase activity, and function in hormone and light stress resistance, developmental processes, and disease resistance. For example, RIN2 and RIN3 encode RING-finger type ubiquitin ligases conferring resistance against *Pseudomonas syringae* infection^23^. Interacting proteins carrying the Znf and RING domains might also have a role in FHB resistance. Proteins A0A3B6NP03 and A0A3B6PLA2 carried the ENTH domain, and proteins carrying this domain are supposed to involve in the initiation of clathrin-coated pit formation in the plasma membrane in endocytosis process. An *Arabidopsis* clathrin assembly protein with a predicted role in plant defence was recently identified^24^. Proteins with DNA-binding domains are involved in the transcriptional regulation of key developmental processes, and proteins with RNA-binding domains also play vital roles in plant gene expression and regulation. Loss of function of these proteins can have a detrimental effect on some critical processes such as photosynthesis and respiration, sometimes leading to lethality^25^. Several such proteins have been found to interact with the PFT protein in this study. Among the interacting proteins, Q96185 is a superoxide dismutase. Rapid production of reactive oxygen species (ROS) after a pathogen attack has been proposed to orchestrate the establishment of different defensive barriers against infection. However, scavenging ROS to an appropriate level in plants is helpful to promote plant development and induce resistance against environmental stresses^26^.

Interacting proteins are more likely to be involved in similar biological functions and processes, and thus are likely to be co-expressed^16^. Thus, quantitative real-time PCR (qRT-PCR) analysis was performed for several tissues, and also under *Fg* stress, to evaluate the expression of the interacting genes. Unlike the PFT expression pattern, most of the interacting genes were highly expressed in the leaf. It should also be noted that all the interacting genes were expressed in the spikes regardless of the expression level. Two genes encoding clathrin assembly proteins, TraesCS6A02G154100 and TraesCS6B02G181800, were particularly expressed in the spikes. Eight genes, including the above two genes encoding clathrin-related proteins, were up-regulated by at least 1.2-fold 12 h after *Fg* inoculation, and eight genes were up-regulated 48 h after *Fg* inoculation. These *Fg*-induced genes seem to be involved in the ubiquitination process, clathrin coat assembly, the oxidation-reduction process, and protein phosphorylation, and might contribute for PFT function.

Based on these findings, a regulatory network of wheat resistance against *Fg* invasion can be constructed using PFT and its interacting proteins. Once *Fg* invasion is signalled, the expression of *PFT* is up-regulated to improve the host’s antifungal ability. Simultaneously, PFT arouse many pathways to act together to confer *Fg* invasion. For example, it regulates the ubiquitination process by interacting with A0A3B6EIL3, A0A3B5YVG0, and W5GEL1, and the possible endocytosis process involved in plant defence by interacting with A0A3B6NP03 and A0A3B6PLA2. It is also involved in the transcriptional regulation of downstream gene expression by interacting with proteins with DNA/RNA-binding domains, and scavenging the ROS increased level due to the *Fg* invasion by interacting with Q96185. However, it should be acknowledged that all methods have their limitations, and validation of these interacting proteins using different methods, such as immunoprecipitation or bimolecular fluorescence complementation in planta is recommended to obtain reliable results. Another aspect to be noted is that, despite the controversial role of PFT in FHB resistance, the proteins identified here are good indicators to understand the mechanism of PFT action.

## Methods

### Plant materials

The highly FHB-resistant wheat cultivar Sumai 3 was used to construct the Y2H library. Plants were grown in a greenhouse under 14/10 h light/dark and 24/15 °C in Nanjing, China. Spikelets at early anthesis were chosen for further *Fg* inoculation as described previously^27^. After 12, 24, and 48 h of inoculation, samples were harvested and frozen immediately in liquid nitrogen, and then stored at −80 °C until further use.

### Construction of the Y2H library

Total RNA was harvested from the spikes of Sumai 3 8 h after *Fg* inoculation and then treated with DNaseI (Takara, Japan) to eliminate contaminated genomic DNA. The cDNA was synthesized with CDS 4 M adapter (Supplemental Table 1) using the SMART™ cDNA Library Construction Kit (Clontech, USA), and then normalized using the TRIMMER-DIRECT cDNA Normalization Kit (Evrogen, USA) according to the manufacturer’s instructions. To eliminate low-molecular-weight cDNA fragments and small DNA contaminants, the cDNA after *Sfi* I digestion (>200 bp) was excised from a 1% agarose gel and purified using the CHROMA SPIN-1000 column (Clontech, USA). The purified cDNA was merged into the pGADT7-SfiI vector (library of prey proteins with the Gal4 activation domain; Clontech, USA) by directional cloning at *Sfi* IA (5′-GGCCATTACGGCC-3′) and *Sfi* IB (3′-CCGGCGGAGCCGG-5′) sites. Dilutions of the transformed mixture (from 1:10 to 1:1000) were spread on LB media (Amp^+^) and incubated at 37 °C until colonies appeared, to calculate the transformation efficiency and the number of primary colonies. Sixteen colonies were randomly selected and amplified by PCR using primer pGADT7-F/R (Supplemental Table 1) to check insert sizes and the recombination rate of the Y2H library using 1% agarose gel electrophoresis. The primary library was retransformed into competent *Escherichia coli*, and the plasmids were then harvested using the Plasmid Maxi Preparation Kit (Qiagen, Germany). The amplified library was stored at −80 °C by adding dimethyl sulfoxide to a final concentration of 7%.

### Cloning and testing bait for toxicity and autoactivation

The sequences of full-length PFT and its two domains (agglutinin and ETX/MTX2) were obtained by PCR using the primers listed in Supplemental Table 1. The fragments were inserted into pGBKT7 vectors individually. To evaluate the effect on yeast growth, pGBKT7-PFT, pGBKT7-PFTa (PFT(agglutinin)), pGBKT7-ETX/MTX2, and pGBKT7 vectors were separately transformed into *S*. *cerevisiae* strain Y2HGold (Clontech, USA), using Yeastmaker Yeast Transformation System 2 (Clontech, USA). The transformed cells were grown in SD/-Trp medium at 30 °C for 3–5 days, and then diluted at different concentrations (OD 600 = 1, diluted from 1 to 1:1000). One microliter of these dilutions was dotted separately on SD/-Trp medium and grown at 30 °C for 3 days. To test the bait PFTa for autoactivation, pGBKT7-PFTa, the negative control pGBKT7, and the positive control pGBKT7-PCL1, which encodes full-length wild-type GAL4 (able to constitutively activate transcription) were transformed into Y190 yeast cells separately. After culturing at 30 °C for 2 days, colonies were copied to a filter paper and transferred to liquid nitrogen for at least 30 s. The papers were placed in a fresh Z buffer/X-gal solution as described in the Yeast Protocols Handbook (PT3024-1) in a clean 150-mm plate and incubated at 30 °C until blue colonies appeared, which indicated self-activation of the plasmid.

### Screening of the Y2H library

Bait vector pGBKT7-PFTa with the Gal4 DNA-binding domain, together with the prey Y2H AD library with the Gal4 activation domain, was co-transformed into the Y190 strain (Clontech, USA) according to the Yeast Protocols Handbook (PT3024-1) and plated on SD/-Trp/-Leu/-His/+30 mM 3-AT medium at 30 °C for 10 days. Colonies with diameter >2 mm were selected as primary interacting proteins for further analysis and then retransferred into SD/-Trp/-Leu liquid medium culturing for 2 days for isolating vectors using the Yeast Plasmid Extraction Kit (Solarbio, China). The vectors were identified by PCR using the specific primers 5′AD and 3′AD, and then sequenced and analyzed using BLAST (https://blast.ncbi.nlm.nih.gov).

### Confirmation and analysis of positive interactions

The primary interacting proteins and the bait pGBKT7-PFTa vectors were co-transformed into Y2HGold yeast cells individually to confirm their interactions. The transformants were grown in SD/-Trp/-Leu, SD/-Trp/-Leu/-His/X-α-Gal, and SD/-Trp/-Leu/-His/-Ade/X-α-Gal media at 30 °C for 3–5 days. Three replicates were carried out. The sequences from the positive interactions were analyzed using the wheat genome database (TGACv1) at Ensemble Plants (http://plants.ensembl.org/Triticum_aestivum/) to identify gene names and GO terms, and Uniprot (https://www.uniprot.org/uniprot/) to identify protein names and function. Homology function in *A*. *tauschii* subsp. *tauschii* was determined using BLAST. The identified protein sequences were further analyzed using the PFAM (http://pfam.xfam.org/) and SMART (http://smart.embl-heidelberg.de/) databases to search their function domains.

### Extraction of RNA and qRT-PCR analysis

Sumai 3 root, stem, leaf, and spikes, as well as spikes treated with *Fg* were used to extract RNA using the Promega RNA Isolation System (Promega, USA). Quantitative real-time PCR analyses were then performed on a Roche (Switzerland) thermal cycler 96 using SYBR Green (Takara, Dalian, China) to detect gene expression. The wheat *tubulin* gene was used as the endogenous control gene, and the primers used for the qRT-PCR are listed in Supplemental Table 1. The qRT-PCR conditions were as follows: 45 cycles at 95 °C for 30 s, 95 °C for 5 s, 60 °C for 20 s, and 72 °C for 10 s, followed by 95 °C for 10 s, 65 °C for 10 s, and 95 °C for 5 s. The relative abundance of each genes was determined by the comparative Ct method^28^. All reactions were performed in triplicate.

## Supplementary information

Supplementary Information

## Data Availability

The datasets generated during and/or analyzed during the current study are available from the corresponding author on reasonable request.
