# Supplementary material for: Yeast two-hybrid screening for proteins that interact with PFT in wheat
Source: Sci Rep. 2019 Oct 29;9:15521. doi: 10.1038/s41598-019-52030-x (PMC6820867; doi:10.1038/s41598-019-52030-x)
Supplement: Supplementary file 1 — Supplementary Information [file 41598_2019_52030_MOESM1_ESM.docx]

**Yeast two-hybrid screening for proteins that interact with PFT in wheat**

**Yi He^1^, Lei Wu^1^, Xiang Liu^2^, Xu Zhang^1^, Peng Jiang^1^ and Hongxiang Ma^1﹡^**

**^1^ Institute of Food Crops, Jiangsu Academy of Agricultural Sciences / Jiangsu Collaborative Innovation Center for Modern Crop Production, Nanjing, China**

**^2^ Tibet Agriculture and Animal Husbandry University, Linzhi, China**

**^﹡^Correspondence:**

**Hongxiang Ma**

**hongxiangma@163.com**


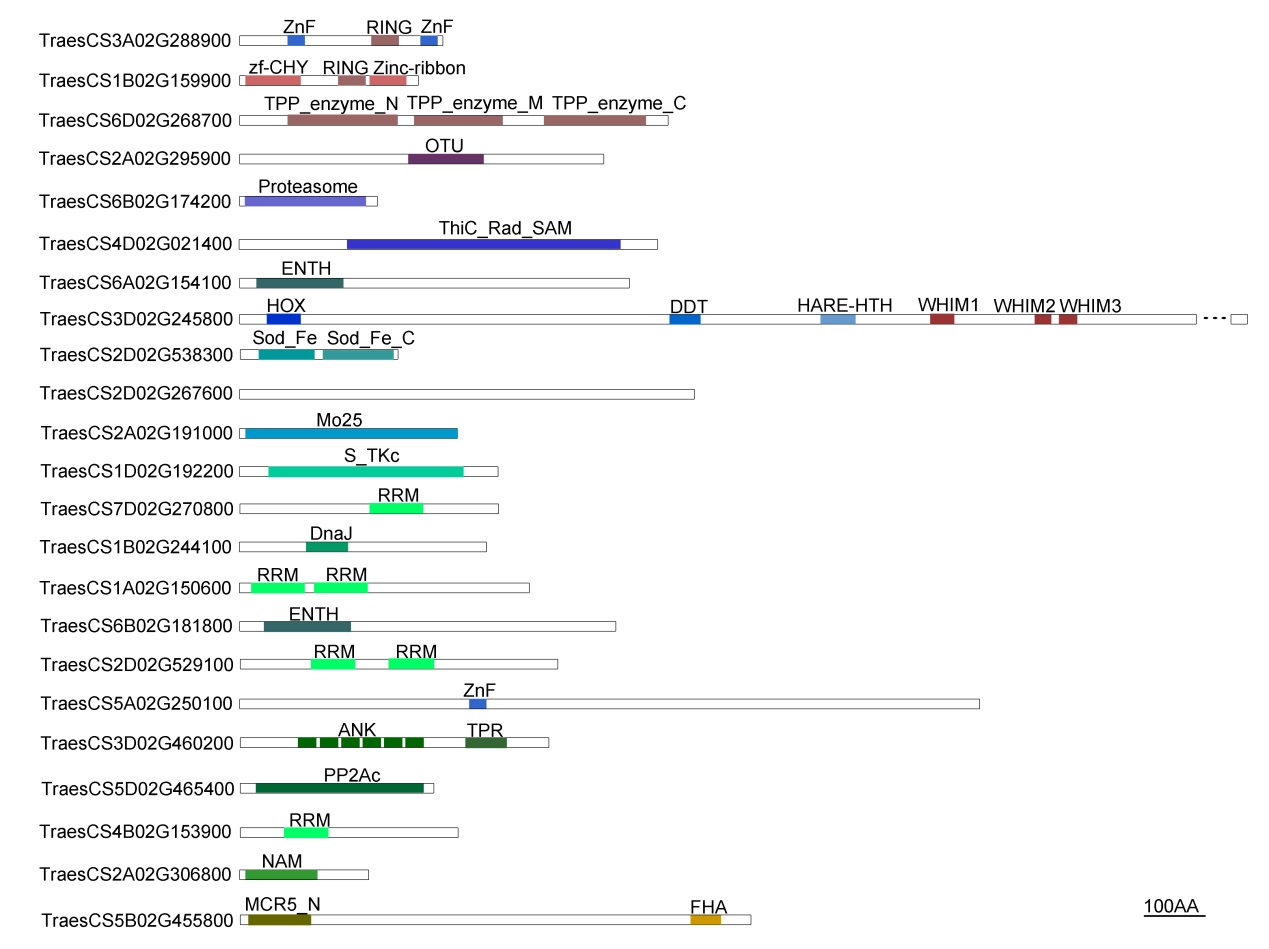


Supplementary Fig. 1. Schematic presentation of the domain structures of the 23 proteins. ZnF, Zinc finger; zf-CHY, CHY-type zinc finger; RING, really interesting gene; Zinc-ribbon, zinc-ribbon finger; TPP_enzyme, thiamine pyrophosphate enzyme; OTU, ovarian tumour; Proteasome, proteasome subunit; ThiC_Rad_SAM, Radical SAM ThiC family; ENTH, Epsin N-terminal homology domain; HOX, homeobox domain; DDT, domain in different transcription and chromosome remodeling factors; HARE-HTH, HB1, ASXL, restriction endonuclease HTH domain; WHIM, WSTF, HB1, Itc1p, MBD9 motif; Sod_Fe, Fe superoxide dismutase; Mo25, Mo25-like; S_TKc, Serine/Threonine protein kinases, catalytic domain; RRM, RNA recognition motif; DnaJ, DnaJ molecular chaperone homology domain; ANK, Ankyrin repeats; TPR, Tetratricopeptide repeats; PP2Ac, Protein phosphatase-2A; NAM, No apical meristem protein; MCRS_N, N-terminal region of micro-spherule protein; FHA, Forkhead associated domain.

Supplemental Table 1. Primers used in this study.

| **Primer name** | **Sequence （5'-->3'）** | **Objective** |
| --- | --- | --- |
| PFT-Y2HF | CCCATATGATGTTTCCGCTGTCAGCTTT | yeast toxicity |
| PFT-Y2HR | CGGGATCCCAGGTGTTCTTCTTTGGTAT | yeast toxicity |
| PFT(Agg)-Y2HF | CCCATATGATGTTTCCGCTGTCAGCTTT | yeast toxicity |
| PFT(Agg)-Y2HR | CGGGATCCTTCAATGCACCGCATTTTTA | yeast toxicity |
| PFT(ETX)-Y2HF | CCCATATGGTTTCTCGAGACATCTATGA | yeast toxicity |
| PFT(ETX)-Y2HR | CGGGATCCCAGGTGTTCTTCTTTGGTAT | yeast toxicity |
| CDS 4M adapter | AAGCAGTGGTATCAACGCAGAGTGGCCGAGGCGGCC(T)4G(T)6C(T)13VN | cDNA synthesize |
| pGB-PFTa-F | AAAAGGCCAATCCGGCCATGTTTCCGCTGTCAGCTTT | autoactivation and screening Y2H AD library |
| pGB-PFTa-R | AAAAGGCCTTAGAGGCCTTCAATGCACCGCATTTTTA | autoactivation and screening Y2H AD library |
| pGADT7-F Primer | GGAGTACCCATACGACGTACC | size of the inserting cDNA fragment |
| pGADT7-R Primer | TATCTACGATTCATCTGCAGC | size of the inserting cDNA fragment |
| 5'AD | CTATTCGATGATGAAGATACC | sequencing |
| 3'AD | AGATGGTGCACGATGCACAG | sequencing |
| TraesCS3A02G288900-RT-F | CGAAAGATTTGAGAAGGGAT | RT-qPCR |
| TraesCS3A02G288900-RT-R | TGGAAGTTCATGTCGCCTAA | RT-qPCR |
| TraesCS1B02G159900-RT-F | CAGTACCACTGCGATGGATG | RT-qPCR |
| TraesCS1B02G159900-RT-R | GCAGTGCAGTACGCTGATGT | RT-qPCR |
| TraesCS6D02G268700-RT-F | CGGTTTGATGATCGTGTGAC | RT-qPCR |
| TraesCS6D02G268700-RT-R | CATGGACCAAAATCCAGACC | RT-qPCR |
| TraesCS2A02G295900-RT-F | GACTCCAATGTGACCGATCC | RT-qPCR |
| TraesCS2A02G295900-RT-R | CCAGATCCCATTCTCCTCAA | RT-qPCR |
| TraesCS6B02G174200-RT-F | CCGACTTCCAGAGGGTGTTC | RT-qPCR |
| TraesCS6B02G174200-RT-R | ATCGGTTGGCAGAAGTATGG | RT-qPCR |
| TraesCS4D02G021400-RT-F | AGATGGTTGGACCAATTTGC | RT-qPCR |
| TraesCS4D02G021400-RT-R | CACCGCTTCCTCTACTGTCC | RT-qPCR |
| TraesCS6A02G154100-RT-F | ACTACCAGCCTCAGCTCCAA | RT-qPCR |
| TraesCS6A02G154100-RT-R | GCCTTGTTTTGAAGGAGCAG | RT-qPCR |
| TraesCS3D02G245800-RT-F | TGCCTAGGATTTCTGCACCT | RT-qPCR |
| TraesCS3D02G245800-RT-R | AAGCGGATCAAAATCGACAC | RT-qPCR |
| TraesCS2D02G538300-RT-F | GCCATTGATGAGGATTTTGG | RT-qPCR |
| TraesCS2D02G538300-RT-R | CCCAGACATCAATTCCCAAC | RT-qPCR |
| TraesCS2D02G267600-RT-F | ACCGAATCGTTGGTTGAGAG | RT-qPCR |
| TraesCS2D02G267600-RT-R | GCACCCCAAGATGAGCTAAG | RT-qPCR |
| TraesCS2A02G191000-RT-F | ACCTGGAGGCAAGGAAAGAT | RT-qPCR |
| TraesCS2A02G191000-RT-R | GGCGAATACATTCCCTCAAC | RT-qPCR |
| TraesCS1D02G192200-RT-F | TCTTGCACCGTGATCTGAAG | RT-qPCR |
| TraesCS1D02G192200-RT-R | TATGCACCCAACTGACCAGA | RT-qPCR |
| TraesCS7D02G270800-RT-F | TCCTGTTGATGACCACCGTA | RT-qPCR |
| TraesCS7D02G270800-RT-R | TTGACAAGCCTCACTTCACG | RT-qPCR |
| TraesCS1B02G244100-RT-F | TGGAGTGGAAAGGACTTGCT | RT-qPCR |
| TraesCS1B02G244100-RT-R | TTAACCTCATGCCCCTCAAG | RT-qPCR |
| TraesCS1A02G150600-RT-F | GCTGGAAATCCTGCTACTGG | RT-qPCR |
| TraesCS1A02G150600-RT-R | CCTAGGCTGCATACCACCAT | RT-qPCR |
| TraesCS6B02G181800-RT-F | ACCCGCGTGACCTACAAT | RT-qPCR |
| TraesCS6B02G181800-RT-R | GCCTTGTTTTGGAGGAGCAT | RT-qPCR |
| TraesCS2D02G529100-RT-F | GTTTGCGCTGTTCGTTTACA | RT-qPCR |
| TraesCS2D02G529100-RT-R | CAATAGAGGGCACGGTCATT | RT-qPCR |
| TraesCS5A02G250100-RT-F | ATGCAAAGTGCAGTGTCAGC | RT-qPCR |
| TraesCS5A02G250100-RT-R | GCGCCTTTTAGCTTTCCTTT | RT-qPCR |
| TraesCS3D02G460200-RT-F | TATTGCGGCTCTCAATGTTGTC | RT-qPCR |
| TraesCS3D02G460200-RT-R | TAAGCCATTGTTGACAGCAG | RT-qPCR |
| TraesCS5D02G465400-3-RT-F | GGTGATATTCACGGGCAGTT | RT-qPCR |
| TraesCS5D02G465400-2-RT-R | GTGACACAGTTTCGACAGAA | RT-qPCR |
| TraesCS4B02G153900-RT-F | TGGTGAGACTGCTGGAAATG | RT-qPCR |
| TraesCS4B02G153900-RT-R | TCTCTTGCTGCTGATGCAGT | RT-qPCR |
| TraesCS2A02G306800-RT-F | CTCTCCCTCCCTTTCTCTACA | RT-qPCR |
| TraesCS2A02G306800-RT-R | AGTGCTGGCACCCACATT | RT-qPCR |
| TraesCS5B02G455800-RT-F | TTCTTTCCATGCACAGCATC | RT-qPCR |
| TraesCS5B02G455800-RT-R | TTGCTAGATGCCAATTGCTG | RT-qPCR |
